# Oxidation destabilizes toxic amyloid beta peptide aggregation

**DOI:** 10.1038/s41598-019-41931-6

**Published:** 2019-04-02

**Authors:** J. Razzokov, M. Yusupov, A. Bogaerts

**Affiliations:** Research Group PLASMANT, Department of Chemistry, University of Antwerp, Universiteitsplein 1, B-2610 Antwerp, Belgium

## Abstract

The aggregation of insoluble amyloid beta (Aβ) peptides in the brain is known to trigger the onset of neurodegenerative diseases, such as Alzheimer’s disease. In spite of the massive number of investigations, the underlying mechanisms to destabilize the Aβ aggregates are still poorly understood. Some studies indicate the importance of oxidation to destabilize the Aβ aggregates. In particular, oxidation induced by cold atmospheric plasma (CAP) has demonstrated promising results in eliminating these toxic aggregates. In this paper, we investigate the effect of oxidation on the stability of an Aβ pentamer. By means of molecular dynamics simulations and umbrella sampling, we elucidate the conformational changes of Aβ pentamer in the presence of oxidized residues, and we estimate the dissociation free energy of the terminal peptide out of the pentamer form. The calculated dissociation free energy of the terminal peptide is also found to decrease with increasing oxidation. This indicates that Aβ pentamer aggregation becomes less favorable upon oxidation. Our study contributes to a better insight in one of the potential mechanisms for inhibition of toxic Aβ peptide aggregation, which is considered to be the main culprit to Alzheimer’s disease.

## Introduction

Although irrelevant for sporadic Alzheimer’s disease (AD), the aggregation and accumulation of amyloid beta (Aβ) peptides in neural tissue is one of the many causes of AD^1^. These amyloid aggregates interact with the cell membrane, increasing the permeability, such as the calcium influx, which leads to the activation of apoptotic signaling pathways in neuronal cells^2,3^. Therefore, self-assembled forms of Aβ peptides are correlated with neuronal cell death. This results in abnormal cognitive functioning in patients with AD^4^. In order to inhibit the aggregation of Aβ peptides, various classes of small molecules and peptides are used, but these drugs either cannot influence the AD progression, or they are still in clinical trials^5,6^.

A number of experimental and computational studies were already performed, aiming at destabilizing the Aβ peptide aggregates. For instance, molecular dynamics (MD) simulations were conducted to study the contribution of the hydrophobic residues Met, Ile, Phe and Val in the stability of Aβ pentamer^7^. The results indicated that these hydrophobic residues play a potential role in the stability of Aβ pentamer. Moreover, in order to investigate the thermodynamics of peptide dissociation in Aβ pentamer, Lemkul *et al*. mutated specific residues of this system^8^. The authors reported that the hydration level around the Asp23-Lys28 salt bridge promotes the Aβ pentamer stability. The impact of oxidation of Met35 residue on the stability of a Aβ_40_ monomer structure (i.e., Aβ monomer consisting of 40 AAs) was studied by Brown *et al*., varying the pH and the salt concentration of the solution, and employing replica exchange MD simulations^9^. Their results showed the reduction of the β-strand content in the structure, invoked by oxidation of the Met35 residue.

A drop in the aggregation rate was also experimentally observed due to Met35 oxidation of Aβ peptides^10,11^. Specifically, in^10^, the posttranslational modification of Met35 into methionine sulfoxide significantly attenuated the aggregation of Aβ_1–42_ and Aβ_1–40_ peptides, thereby reducing their neurotoxicity. The authors proposed that the oxidation of Met35 can be an example oxidative process, which acts advantageously to slow down the progression of AD^10^. The characteristics of Aβ_1–40_ peptide aggregation before and after oxidation of Met35 were studied in^11^ by Fourier transform ion cyclotron resonance mass spectrometry. The results showed that the formation rate of an Aβ_1–40_ trimer and tetramer considerably reduced after oxidation of Met35, in comparison to the native Aβ_1–40_ peptide. The solid-state NMR data demonstrated that methylation of AAs (Leu17, Phe19, Gly37 and Val39) on Aβ_1–40_ structure efficiently inhibited fibril formation^12^. The ring structure modifications of Phe19 resulted in enhanced fibrillation kinetics and reduced toxicity of Aβ_1–40_ peptides^13^.

Furthermore, applying cold atmospheric plasma (CAP) as an oxidation source, Bayliss and coworkers^14^ treated amyloid aggregates for 2, 4, 6 and 8 s in aqueous solution. Their results showed that 2 s of treatment with CAP already led to a significant reduction of the amyloid aggregates. They attributed this effect to chemical modifications in the structure, caused by CAP. Indeed, CAP produces a cocktail of reactive oxygen and nitrogen species (RONS), and the degradation of amyloid aggregates probably occurs upon oxidation by these RONS. However, the molecular level mechanism is far from fully understood. This explains the need for studying the influence of oxidation, in various degrees, on the stability of the Aβ structure. Up to now, there is no computational evidence on the effect of oxidation on the structure and stability of amyloid aggregates.

Thus, in this paper we perform MD simulations to study the effect of oxidized residues on the stability of Aβ aggregates, taking Aβ pentamer as a model system. We apply the umbrella sampling (US) method^15,16^ to estimate the interaction between the monomers of the Aβ aggregate^8,17,18^. As in our previous studies^19,20^, we consider different oxidation states (i.e., 3%, 9% and 15%), by modifying various AAs according to their reported reactivity^21^. We also calculate the potential mean forces to determine the dissociation free energy of one of the monomers of the Aβ structure upon increasing oxidation degree. The aim of this study is to reveal the mechanism of Aβ fibril destabilization at the molecular level, upon increasing oxidative stress.

## Computational Details

### Simulation setup

**Figure 1:**
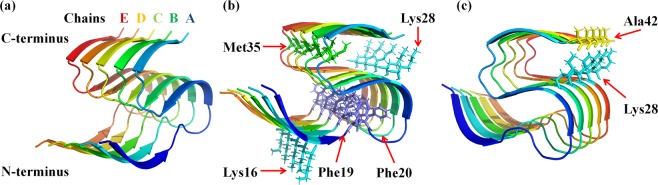
Cartoon view of the Aβ_11–42_ pentamer structure. (**a**) Each chain of the Aβ_11–42_ pentamer structure with C- and N-terminal ends is depicted with a different color. (**b**) AA residues of the Aβ pentamer structure that are modified to create the oxidized structures; oxidation of these AAs occurs in each of the chains (thus five Met35, five Phe19 and Phe20, five Lys16 and Lys28 residues are depicted in licorice view). (**c**) AAs of the Aβ_11–42_ pentamer structure (i.e., Lys28 (cyan) and Ala42 (yellow)) that form a salt bridge in each chain.

We performed MD simulations in order to elucidate the stability of native and oxidized Aβ pentamer at the molecular level. The simulations were carried out using the GROMACS^22^ program package (version 5.1), applying the GROMOS 45a3 force field^23^. We employed the solid-state NMR structure of Aβ_11–42_ pentamer (see Fig. 1) obtained from the Protein Data Bank (ID: 2MXU)^24^. In this structure 10 residues in the N-terminal do not maintain a stable conformation and therefore are not relevant to further aggregation. The N and C terminal ends of each peptide are acetylated and capped with an amide group, respectively.

The native structure of Aβ pentamer (i.e., Aβ_11–42_ pentamer, see Fig. 1a) is placed in a triclinic box, spacing the atoms at least 1.1 nm from the boundaries of the simulation box. Subsequently, the box is filled with an SPC water model^25^ surrounding the Aβ pentamer structure and 0.1 M of NaCl was added to the system to mimic the physiological environment. Besides the native Aβ pentamer, we created three oxidized structures, called OX1, OX2 and OX3, which contain 3, 9 and 15% of oxidized residues, respectively. These oxidized systems were prepared using a web server Viena-PTM 2.0^26^ by replacing the residues of the native Aβ pentamer with oxidized ones. The force field parameters of the oxidized residues were obtained from^27^. These oxidized systems were also placed in a simulation box filled with water, applying the above mentioned steps.

The four different model systems (i.e., native, OX1, OX2 and OX3) were energy minimized with the steepest descent algorithm. Subsequently, a 50 ps equilibration run was performed employing the NVT ensemble (i.e., a system with constant number of particles N, volume V and temperature T), applying the position restraint to the heavy atoms of Aβ pentamer. Next, a 250 ns production run was conducted using the NPT ensemble (i.e., a system with constant number of particles N, pressure P and temperature T) in the absence of a restraint. The simulations were carried out at 310 K and 1 bar, employing the Nose-Hoover thermostat^28^ with a coupling constant of 0.5 ps and the isotropic Parrinello-Rahman barostat^29^ with a compressibility and coupling constant of 4.5 × 10^−5^ bar^−1^ and 2 ps, respectively. A 1.0 nm cut-off distance was applied for the van der Waals interactions. The long range electrostatic interactions were described by the particle mesh Ewald (PME) method^30^. The production run trajectory was used for data collection, i.e., to calculate the root mean square deviation (RMSD)^31^, the solvent accessible surface area (SASA)^32^ and the side chain hydrogen bonds between the neighboring peptides. For the computation of the secondary structural changes of Aβ pentamer, we used the secondary structure assignment program STRIDE^33^, by averaging the data of the MD trajectory acquired from the last 50 ns. The pymol visualizing tool was used to prepare images in this study^34^.

### Umbrella sampling (US)

The starting structure of Aβ pentamer in our US simulations was extracted from the final frame of the production run. The Aβ pentamer structure was placed in a rectangular box and the size of the box was chosen adequately for pulling the monomer out of the Aβ pentamer structure along the *z-*axis. After salination of the system with 0.1 M of NaCl, the system was equilibrated implementing the above mentioned steps (see previous section). Subsequently, applying an external force, the center of mass (COM) of chain A (see Fig. 1a) was pulled along the *z*-axis and chain B was restrained and used as a reference for the pulling simulation. The external force induces displacement of the peptide (chain A in our case) in the simulated system and it allows to calculate the energy in a path dependent manner, i.e., along the reaction coordinate. The pulling simulation lasted for 500 ps, applying 1000 kJ/(mol * nm^2^) spring constant with a pulling rate of 0.01 nm/ps. We extracted 50 windows, calculating the distance between the COM of chain A and B, each separated by 0.1 nm along the *z*-axis. Each umbrella window was then equilibrated for 100 ps, followed by 20 ns of the US simulations. The output files generated from the US simulations were analyzed employing the weighted histogram analysis method (WHAM)^35^ to calculate the potential mean force (PMF). The error associated with the PMF was estimated employing the bootsrapping method^36^.

### Selection of AAs for the creation of the oxidized structures

Takai *et al*. have determined the chemical modification of AAs by treating them individually with CAP and they reported the following order for the reactivity of the AAs (in order of decreasing reactivity): Met → Cys → Trp → Phe → Tyr → the rest of the AAs^21^. Based on these results, and because the native Aβ pentamer structure does not contain Cys, Trp and Tyr, we decided to modify Met35, Phe19, Phe20, Lys16 and Lys28 of each chain in the Aβ pentamer. Lysine residues were chosen because one of these residues, particularly Lys28, is involved in the formation of the Lys28-Ala42 salt bridge (see Fig. 1c), and we want to study the influence of the disruption of this bridge on the stability of Aβ pentamer, through oxidation of Lys28.

Specifically, we changed Met35 to methionine sulfoxide, Phe19 and Phe20 to 3,4- dihydroxyphenylalanine and Lys16 and Lys28 to allysine (see Table 1). In this way, we created three different oxidation degrees of Aβ pentamer, assumed to happen upon oxidation by CAP, i.e., OX1, OX2 and OX3, as given in Table 1.

**Table 1:** Modified AAs to create the oxidized Aβ pentamer structures.

Oxidation	Modified AAs in Aβ	Native AA	Oxidized AA	Oxidation percentage (%)
OX1	Met35			3
OX2	OX1 + Phe19, Phe20			9
OX3	OX2 + Lys16, Lys28			15

The choice of the specific AAs for modification is based on the results of ^21^.

## Results and Discussion

**Figure 2:**
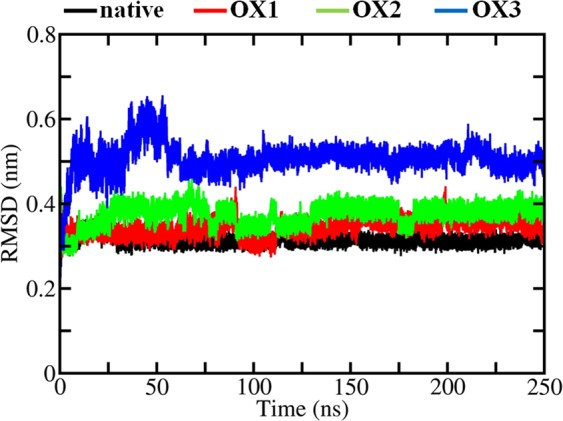
RMSD of the backbone of the native, OX1, OX2 and OX3 Aβ pentamer structures.

Figure 2 illustrates the time evolution of the RMSD of the backbone of the native and oxidized Aβ pentamer structures.

**Figure 3:**
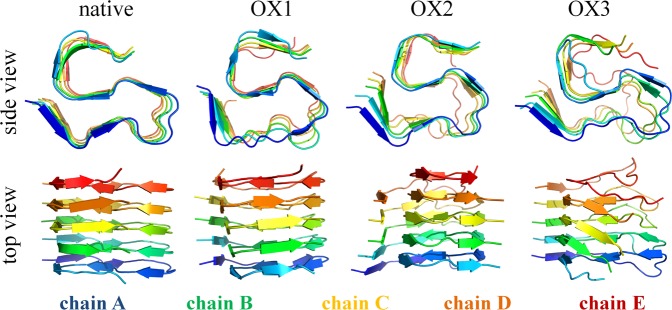
Last snapshots of the 250 ns MD simulations, showing the structures of native Aβ pentamer, OX1, OX2 and OX3, both in side and top view, to illustrate the conformational changes of the native and oxidized Aβ pentamer structures.

The RMSD of the native Aβ pentamer structure is the most stable with the lowest fluctuations. In the case of OX1, the RMSD fluctuations slightly increase. Oxidation of the Met35 residue leads to small destabilization in the structure (cf. also Fig. 3 below). A further increase of the oxidation level leads to even higher fluctuations in the RMSD (see OX2 and OX3 in Fig. 2). The calculated average value of the RMSD increases upon increasing oxidation (see Table S1). This indicates that the oxidized structures become more flexible, affecting their conformations. However, the salt bridge Lys28-Ala42 (see Fig. 1c) in the native, OX1 and OX2 structures maintains its integrity in the Aβ pentamer (see Fig. 3), whereas in OX3, the mobility of the C-terminal in each chain increases after oxidation of Lys28, leading to a disruption of the salt bridge interaction. This in turn results in a higher solvent accessibility and conformational changes in the system (cf. the results given in Tables S1 and S2). Indeed, the SASA analysis confirms that the overall solvent accessibility rises upon increasing oxidation of the Aβ pentamer structure (see Table S1).

To support our RMSD and SASA results, we performed secondary structure analysis to obtain detailed information on the conformational changes. The main conformation of the native structure consists of β-sheets, i.e., 73.3.% (Table 2). The content of these β-sheets reduces in the OX3 structure, turning them into other conformations, e.g., to coil structures (see Table 2). The fraction of β-sheet conformation in OX3 decreases by 9.3% compared to the native structure, which is most likely due to the absence of the salt bridge. In OX1 and OX2 we observed the increased β-sheet content. Nevertheless, the flexibility and SASA of these structures are still higher than in the native case, whereas the hydrogen bonds between the chains are lower (see Table S1). Thus, these results overall indicate that despite the increase of the β-sheet content, oxidation in general leads to complex conformational changes, thereby resulting in a destabilization of the Aβ pentamer.

**Table 2:** Secondary structure analysis of the native and oxidized Aβ pentamer structures.

System	β-sheet	β-bridge	Turn	Coil	α-, 3- and 5-helix
native	73.3	2.1	9.7	14.9	0
OX1 (3%)	80.0	0.8	7.3	11.9	0
OX2 (9%)	74.7	0.8	8.7	15.8	0
OX3 (15%)	64.0	1.2	12.6	22.2	0

The values denote the relative occurrence (in %) of the various conformations.

Note that the hydrogen bonds between the chains are one of the factors that strengthen the inter-peptide interactions. Hence, a decrease in the number of hydrogen bonds results in weakening of the binding energy between the chains of Aβ pentamer.

**Figure 4:**
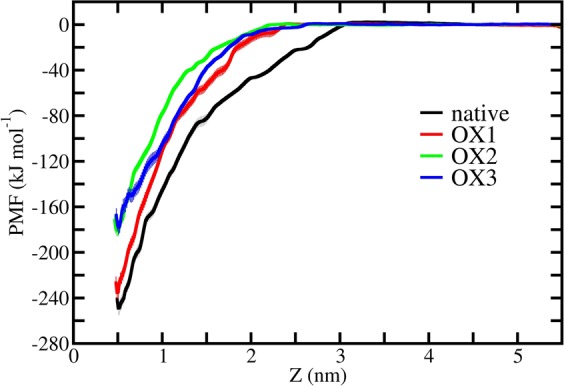
PMF profiles of the native and oxidized Aβ pentamer structures. The errors associated with sampling are presented in pale color.

In order to quantitatively estimate the interactions between the chains of the native and oxidized structures, we performed US simulations to determine the dissociation free energies of the terminal peptides (i.e., energy between chain A and B). The dissociation free energy profiles help us to draw conclusions about the stability of the Aβ pentamer structures. Figure 4 illustrates the PMF profile of the terminal chain A pulled against chain B. The difference between the minimum and maximum values of the PMF gives us the dissociation (or binding) free energy.

It is clear from Fig. 4 that the terminal chain A of the native Aβ pentamer totally disintegrates at 3.1 nm distance, whereas in the oxidized structures it can dissociate even at shorter distances, i.e., 2.40, 2.27 and 2.61 nm in the cases of OX1, OX2 and OX3, respectively. This indicates that the interaction between chain A and B weakens upon increasing oxidation, and at the same time, the PMF values also decrease. The calculated dissociation free energies are found to be −248.93, −235.39, −181.05 and −178.17 kJ/mol for native, OX1, OX2 and OX3, respectively (see Table 3). Hence, a higher oxidation level leads to a lower binding free energy, which is most pronounced in the case of OX3. The small difference in binding free energy between the OX2 and OX3 system (i.e., 2.88 kJ/mol) is probably due to the twist of the OX3 structure (see Fig. 3), which leads to a formation of extra hydrogen bonds between chains A and C, although their relative occurrence is quite low (see Table S4).

**Table 3:** Binding free energies ∆G for native and oxidized Aβ pentamer structures.

System	∆G (kJ/mol)	∆∆G (kJ/mol)
native	−248.93	—
OX1 (3%)	−235.39	+13.54
OX2 (9%)	−181.05	+67.88
OX3 (15%)	−178.17	+70.76

∆∆G represents the loss of energy compared to the native case.

Thus, we can conclude that oxidation of Aβ pentamer leads to a higher flexibility and more conformational changes in the structure, thereby increasing the solvent accessibility. This is more obvious in the case of OX3 due to the disruption of the salt bridge interaction. Moreover, oxidation results in a decrease of the inter-peptide binding free energy, eventually destabilizing the Aβ aggregation process.

Bayliss and co-workers showed that 2 s of treatment with CAP already significantly reduces the amyloid aggregates^14^. They also investigated the effect of heat (80 °C) and gas flow (without discharge) and the combination of both, and found that these physical treatments have little impact on the morphology of the amyloid aggregates. Instead, the chemical effects caused by CAP-generated ROS were found to play a vital role in degradation of the amyloid aggregates^14^. Our simulation results are in qualitative agreement with this experimental observation, and they can explain the underlying mechanisms. Indeed, we find that (CAP-induced) oxidation of Aβ pentamer leads to a lower inter-peptide binding free energy, eventually resulting in degradation of the amyloid aggregates. We also checked the effect of heat (80 °C) on the flexibility, the solvent accessibility, the conformation and the binding free energy between chains A and B of the Aβ pentamer (see Tables S1 and S2 and Fig. S2). In general, we can conclude that this high temperature does not strongly influence the binding free energy, despite the elevated fluctuations and conformational changes observed in the system. The calculated binding free energy is found to be −239.4 kJ/mol (see Fig. S2), which is quite close to the value of OX1 (cf. Table 3). Thus, we can conclude that the salt bridge plays an essential role in the stability of the Aβ pentamer, and disruption of this interaction (through e.g., oxidation by CAP, or ROS created in another way) leads to destabilization, resulting eventually in degradation of the amyloid aggregates. It has to be mentioned that in reality, chemical reactions between (CAP-generated) ROS and amyloid aggregates take place, resulting in oxidation of the AAs or even breakage of the peptide bonds^14^. This can lead to even higher damages in the amyloid aggregates. Chemical reactions cannot be studied by our non-reactive MD simulations, but our US simulations help to gain valuable information about the consequences of oxidation in the Aβ pentamer stability.

## Conclusion

We studied the effect of oxidation on the stability of Aβ pentamer, employing MD and US simulations. The results unambiguously demonstrate that a low and moderate degree of oxidation (OX1 and OX2) have insignificant impact on the conformation and stability, whereas a higher oxidation degree (OX3), i.e., leading to disruption of the salt bridge, yields a considerable disturbance of the structure. This is a hallmark for the possible inhibition of Aβ pentamer aggregation. In this regard, the salt bridge plays a key role in the integrity and stability of the Aβ pentamer structure.

Our results are in qualitative agreement with experiments where a CAP source was used to eliminate amyloid aggregates, and they can explain the underlying mechanisms. Indeed, CAP-generated reactive oxygen species (ROS) cause oxidation in the amyloid aggregates through chemical modifications (i.e., not through physical effects like heat and gas flow), ultimately leading to degradation of the aggregates.

Thus, CAP-induced oxidation could be beneficial to eliminate toxic Aβ aggregates that cause several diseases, including AD. Based on these considerations, we suggest to examine the effects of CAP (or plasma-treated liquids^37^) on these diseases by means of *in vivo* experiments. In addition, the computational investigations would also be useful to study more complex conformations of highly neurotoxic amyloid fibrils given in literature^38–41^.

Our study is particularly important for the potential application of CAP, but it is also of more general interest to other oxidation-inducing therapies.

## Supplementary information

Supporting info
