# Supplementary material for: Oxidation destabilizes toxic amyloid beta peptide aggregation
Source: Sci Rep. 2019 Apr 2;9:5476. doi: 10.1038/s41598-019-41931-6 (PMC6445117; doi:10.1038/s41598-019-41931-6)
Supplement: Supplementary file 1 — Supporting info [file 41598_2019_41931_MOESM1_ESM.docx]

**Oxidation destabilizes toxic amyloid beta peptide aggregation**

**J. Razzokov,* M. Yusupov and A. Bogaerts**

*Research Group PLASMANT, Department of Chemistry, University of Antwerp, Universiteitsplein 1, B-2610 Antwerp, Belgium*

E-mail: jamoliddin.razzokov@uantwerpen.be

**MD simulations**

We performed MD simulations for 250 ns after energy minimization and subsequent equilibration simulations. The last 50 ns trajectory was used to analyze the average values of the backbone root mean square deviation (RMSD), the solvent accessible surface area (SASA) and the hydrogen bonds between neighboring chains (Table S1), as well as the secondary structure (Table S2) and the relative occurrence of hydrogen bonds between the chains A and C in OX3 system (Table S3).

***Table S1.*** *Average RMSD of the backbone (nm), SASA (nm­^2^) and number of hydrogen bonds between the chains, with associated standard deviations, for the native and three oxidized Aβ pentamer structures, as well as the native structure after heat treatment.*

| Systems | RMSD | SASA | h-bonds |
| --- | --- | --- | --- |
| native | 0.31±0.01 | 73.55±1.17 | 31.09±2.42 |
| OX1 (3%) | 0.36±0.01 | 74.63±1.33 | 27.32±2.28 |
| OX2 (9%) | 0.39±0.02 | 75.79±1.31 | 26.64±2.42 |
| OX3 (15%) | 0.52±0.03 | 76.89±1.29 | 23.70±2.35 |
| native 80 °C | 0.47±0.16 | 77.30±1.41 | 25.12±2.63 |

***Table S2.*** *Secondary structure analysis for the native and three oxidized Aβ pentamer structures, as well as the native structure after heat treatment.*

| Systems | β-sheet | β-bridge | Turn | coil | α-, 3- and 5-helix |
| --- | --- | --- | --- | --- | --- |
| native | 73.3 | 2.1 | 9.7 | 14.9 | 0 |
| OX1 (3%) | 80.0 | 0.8 | 7.3 | 11.9 | 0 |
| OX2 (9%) | 74.7 | 0.8 | 8.7 | 15.8 | 0 |
| OX3 (15%) | 64.0 | 1.2 | 12.6 | 22.2 | 0 |
| native 80 °C | 70.0 | 2.0 | 10.0 | 18.0 | 0 |

***Table S3.*** *Hydrogen bonds formed between chains A and C in OX3 system. Data is collected from the analysis of 10000 frames of the last 50 ns.*

| Number of h-bonds | Relative occurrence (%) |
| --- | --- |
| 0 | 99.35 |
| 1 | 0.61 |
| 2 | 0.04 |

To support our RMSD results, we performed a principle component analysis (PCA) for the native and oxidized Aβ pentamer structures, where we studied the collective motion of the alpha carbons by plotting the projection of the first eigenvector (representing the direction of the highest motion) versus the projection of the second eigenvector (representing the second highest motion). In other words, the PCA results show the total phase space that each Aβ pentamer structure is able to occupy. The PCA results are presented in Figure S1. In general, we can conclude that the oxidation leads to an increase of the total phase space occupied by the structure, thereby resulting in an increased flexibility.


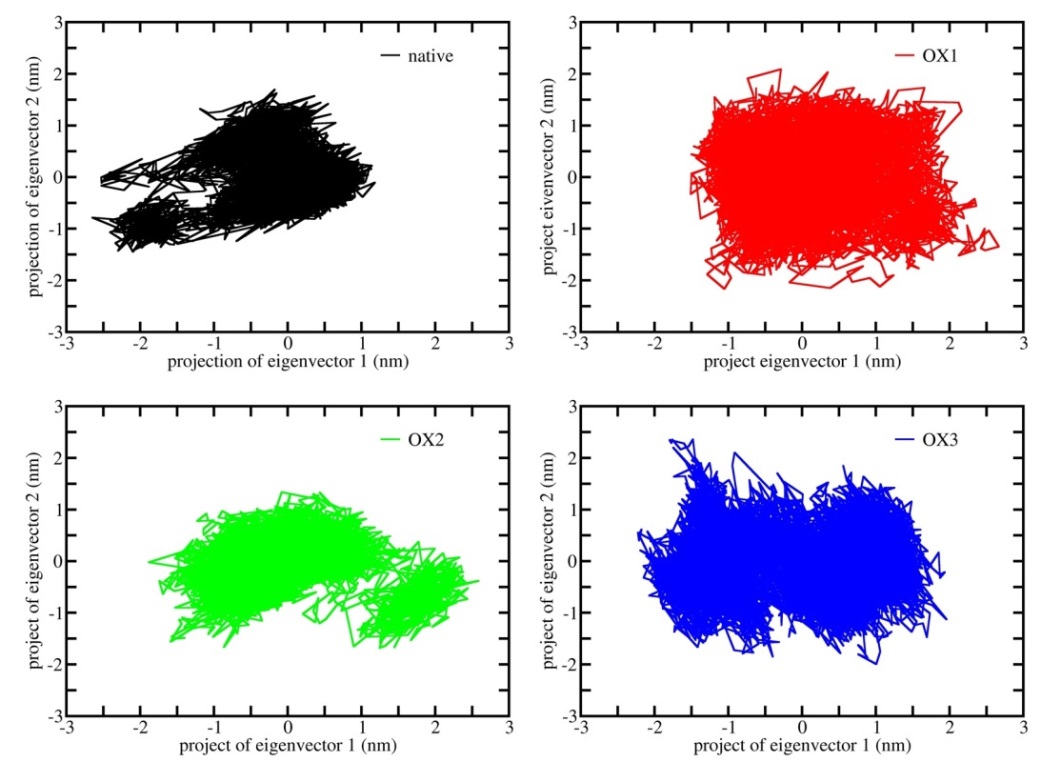


***Figure S1.*** *PCA results obtained for the native and oxidized Aβ pentamer structures, collecting the data from the last 50 ns of MD simulations.*

*
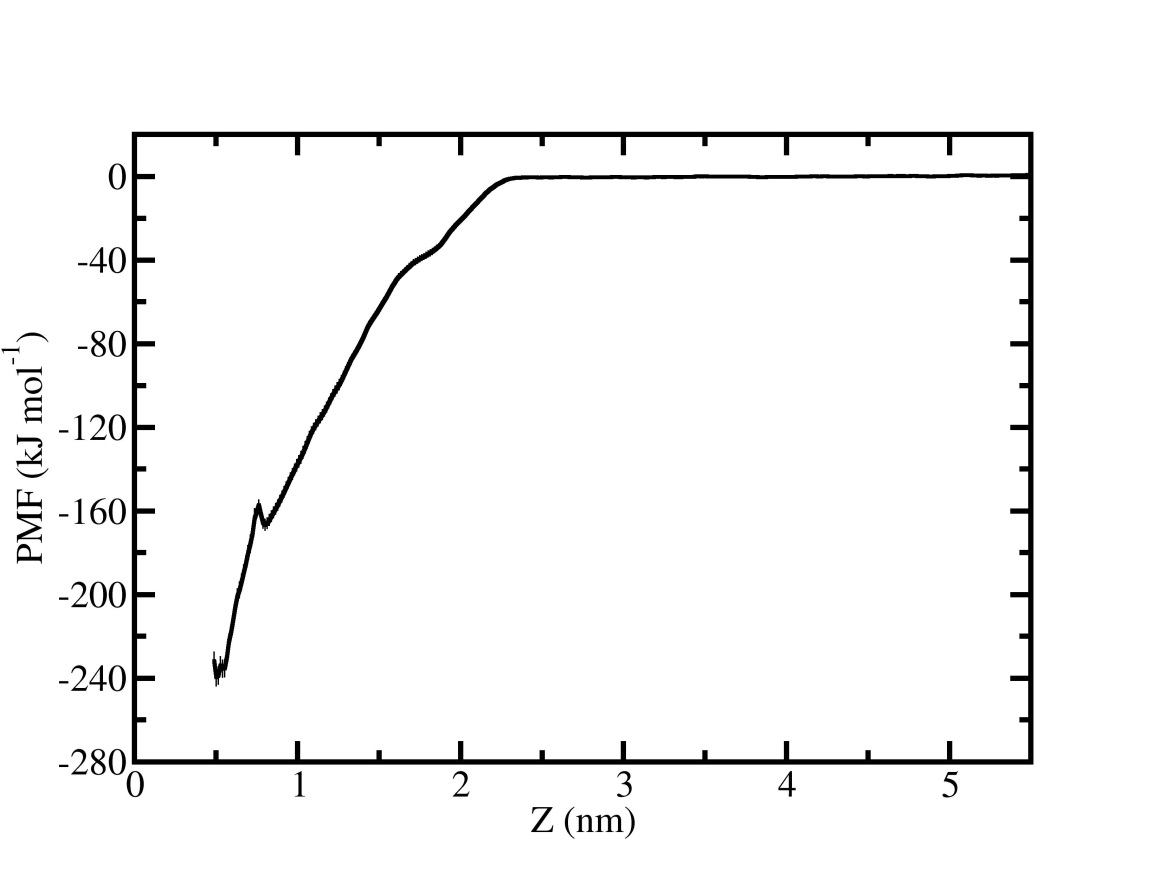
*

***Figure S2.*** *PMF profile of the native Aβ pentamer structure at 80*°C*.*

***
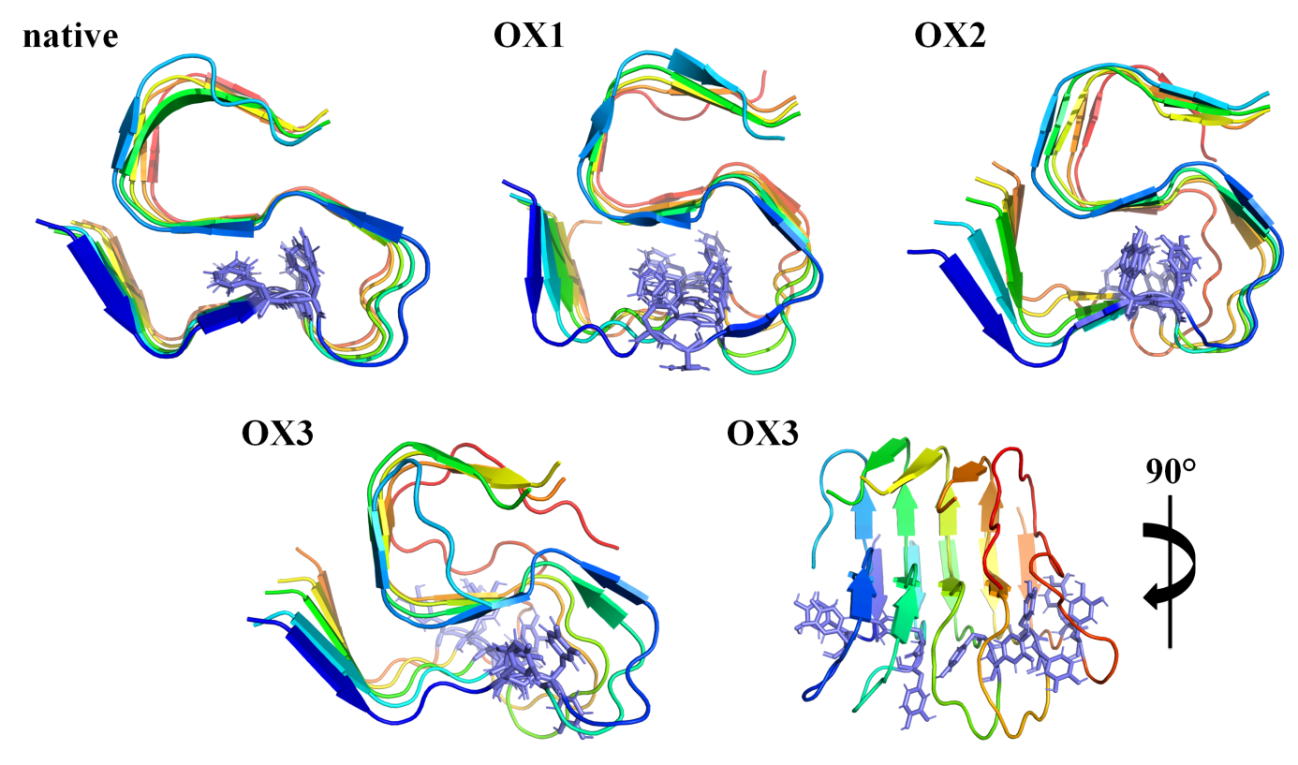
***

***Figure S3.*** *Arrangement of Phe19 and Ph20 in the hydrophobic core of native, OX1, OX2 and OX3 system.*
